# FXR Agonist INT-747 Upregulates DDAH Expression and Enhances Insulin Sensitivity in High-Salt Fed Dahl Rats

**DOI:** 10.1371/journal.pone.0060653

**Published:** 2013-04-04

**Authors:** Yohannes T. Ghebremariam, Keisuke Yamada, Jerry C. Lee, Christine L. C. Johnson, Dorothee Atzler, Maike Anderssohn, Rani Agrawal, John P. Higgins, Andrew J. Patterson, Rainer H. Böger, John P Cooke

**Affiliations:** 1 Division of Cardiovascular Medicine, Stanford University, Stanford, California, United States of America; 2 Department of Pediatrics, Stanford University, Stanford, California, United States of America; 3 Institute of Clinical Pharmacology and Toxicology, University Medical Center Hamburg-Eppendorf, Hamburg, Germany; 4 Division of Anesthesia, Stanford University, Stanford, California, United States of America; 5 Division of Pathology, Stanford University, Stanford, California, United States of America; The Chinese University of Hong Kong, Hong Kong

## Abstract

**Aims:**

Genetic and pharmacological studies have shown that impairment of the nitric oxide (NO) synthase (NOS) pathway is associated with hypertension and insulin-resistance (IR). In addition, inhibition of NOS by the endogenous inhibitor, asymmetric dimethylarginine (ADMA), may also result in hypertension and IR. On the other hand, overexpression of dimethylarginine dimethylaminohydrolase (DDAH), an enzyme that metabolizes ADMA, in mice is associated with lower ADMA, increased NO and enhanced insulin sensitivity. Since DDAH carries a farnesoid X receptor (FXR)-responsive element, we aimed to upregulate its expression by an FXR-agonist, INT-747, and evaluate its effect on blood pressure and insulin sensitivity.

**Methods and Results:**

In this study, we evaluated the in vivo effect of INT-747 on tissue DDAH expression and insulin sensitivity in the Dahl rat model of salt-sensitive hypertension and IR (Dahl-SS). Our data indicates that high salt (HS) diet significantly increased systemic blood pressure. In addition, HS diet downregulated tissue DDAH expression while INT-747 protected the loss in DDAH expression and enhanced insulin sensitivity compared to vehicle controls.

**Conclusion:**

Our study may provide the basis for a new therapeutic approach for IR by modulating DDAH expression and/or activity using small molecules.

## Introduction

Salt-sensitivity (SS), dysregulated changes in blood pressure (BP) in response to salt-intake, is principally influenced by genetics, diet, age and socio-economic factors and is strongly associated with increased cardiovascular (CV) risk and mortality [1], [2]. One of the outcomes of high-salt consumption by salt-sensitive individuals is an increase in BP that may be refractory to multiple antihypertensive therapies [3]. Elevated BP increases the risk of coronary heart disease and stroke [2], [4]. In addition, high salt intake is associated with renal calculi and other kidney diseases [4], [5].

In this regard, the Dietary Approaches to Stop Hypertension (DASH) study demonstrated a significant reduction in BP by reducing salt-intake and/or by consuming the “DASH diet” [6]; a good source of dietary nitrates which can be converted to nitric oxide (NO) in vessel walls [7]. Further evidence that endogenous NO regulates BP in humans comes from studies of the T-786C polymorphism of the eNOS gene promoter, which reduces eNOS activity, impairs endothelial function and increases the risk for essential [8] and salt-induced hypertension (HTN) [9], [10]. Endothelium-derived NO is thought to play a crucial role in vascular and metabolic homeostasis [11], [12]. In pre-clinical models, genetic disruption or pharmacological inhibition of NOS causes HTN and insulin resistance (IR) [13]–[15].

The IR syndrome is manifested by compensatory hyperinsulinemia and is associated with increased CV risk [16], [17]. The association of IR with CV risk may be mediated by its adverse effect on a variety of endothelial functions including those related to vascular reactivity, structure, inflammation and thrombosis [18]. In addition to its contribution to these endothelial dysfunctions, a reduction in NO may also play a role in the development of IR [15]. NO modulates insulin-mediated glucose disposal as well as reactivity of vessels in insulin-sensitive tissues [19]. Furthermore, mice with gene disruption of either endothelial or neuronal NOS exhibit IR [13], [20], possibly due to the consequent reduction in microvascular recruitment and/or muscle glucose uptake in response to insulin. Similarly, excessive inhibition of NOS by its endogenous inhibitor, asymmetric dimethylarginine (ADMA), is associated with IR [21]. In addition, pre-clinical and human data suggest that salt sensitive HTN (which is also associated with IR) may be mediated in part by ADMA [22], [23].

Plasma levels of ADMA are highly dependent upon the activity of the enzyme dimethylarginine dimethylaminohydrolase (DDAH) [24]–[26]. DDAH is present in all nucleated cells in one of two isoforms [25]. DDAH is highly sensitive to oxidative stress [27], and a number of CV risk factors impair its activity, leading to elevated ADMA levels and impairment of the NOS pathway [25]. The DDAH1 transgenic mouse expresses greater DDAH activity and has reduced plasma and tissue levels of ADMA [28]. As a result, NOS activity is upregulated, plasma and urinary nitrogen oxides are increased, and vascular resistance and mean arterial pressure (MAP) are reduced [29]. Intriguingly, we have found that the DDAH transgenic mouse has greater insulin sensitivity [28]. Accordingly, we hypothesized that pharmacological upregulation of DDAH expression might enhance insulin sensitivity. Therefore, we studied the effect of a farnesoid x receptor (FXR) agonist, Obeticholic Acid (INT-747), on tissue DDAH expression and insulin sensitivity in an animal model of salt-sensitive hypertension [30], [31]. The rationale for the use of an FXR agonist in this study is based on the presence of putative FXR response element in the DDAH1 promoter [32] and on previous studies that demonstrated upregulation of DDAH expression using this approach [32]–[34]. FXR belongs to the family of nuclear receptors essential in the regulation of lipids, glucose and bile acid. In this study, we found that INT-747 upregulated liver DDAH1 expression and enhanced insulin sensitivity in Dahl rats.

## Materials and Methods

### Animals and Experimental Design

Dahl salt-sensitive (SS/JrHsd) male 6-weeks old rats and low (0.49% sodium chloride (NaCl)) and high-salt (8% NaCl) Teklad Custom Research diets were all purchased from Harlan Laboratories (Indianapolis, IN). INT-747 was kindly provided by Intercept Pharmaceuticals (Perugia, Italy). ADMA and nitrogen oxides (NOx) were measured using kits from DLD Diagnostika (Hamburg, Germany) and Assay Designs (Ann Arbor, MI) respectively. Urinary albumin and creatinine measurement kits were purchased from Exocell (Philadelphia, PA). Methylcellulose and glucose were purchased from Sigma (St Louis, MO). Plasma insulin was measured using the ultra-sensitive rat insulin ELISA kit (Crystal Chem, Inc; Downers Grove, IL). Tail-cuff blood pressure measurements were using the BP-2000 blood pressure analysis system (Visitech Systems Inc; Apex, NC). Western blot antibodies were purchased from suppliers described in the text. Histological sectioning as well as standard H&E and Trichrome staining was performed at Stanford University (Department of Comparative Medicine).

### Ethics Statement

The in vivo study adhered with the Guide for the Care and Use of Laboratory Animals published by the US National Institutes of Health (NIH Publication, 8th Edition, 2011). Isoflurane inhalation (2%) was used in all the procedures involving anesthesia, prior to performing these procedures, and was frequented as necessary to assure adequate depth of anesthesia during procedures as described below. The adequacy of the depth of anesthesia was assured prior to procedures and maintained during procedures by monitoring responsiveness to stimuli (such as footpad-pinch), observing color changes to the ears and foot pads and monitoring vital signs such as heart rate and breathing pattern. At the end of the study period, the animals were sacrificed by cervical dislocation under anesthesia following the American Veterinary Medical Association (AVMA) guidelines on euthanasia. The study was approved by Stanford's Institutional Animal Care and Use Committee (IACUC; permit # 24045).

### High salt-induced Hypertension

Initially, all animals (at 6-weeks age) were placed on a standard rodent diet for a week. Baseline blood and urine samples were collected and basal blood pressure (BP) was measured prior to grouping the animals. Subsequently, the animals were randomized into low (LS; n = 9) or high salt (HS) diet groups. Hypertension was induced in the HS group by daily high-salt diet feeding and the group was subdivided to receive one of two doses of INT-747: low dose (10 mg/kg/day; n = 15) or high dose (30 mg/kg/day; n = 15) in 1% methylcellulose; or vehicle (1% methylcellulose in distilled water; n = 15) orally everyday for 6 weeks. In parallel, the LS group also received 1% methylcellulose. BP was measured weekly for the duration of the study as described below.

### Hemodynamic measurements

**Figure 1:**
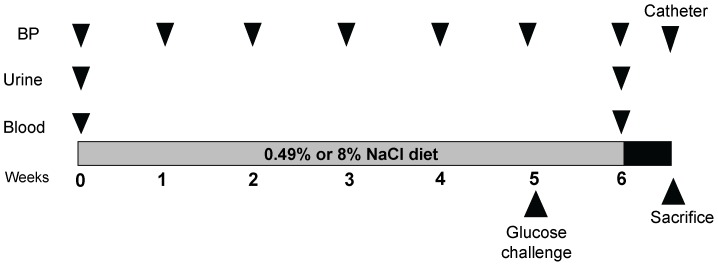
Schematic showing the time frame and experimental protocol followed to induce hypertension by high-salt feeding. Measurements period of BP, glucose challenge, urinary collection and blood draw are also shown. BP = blood pressure.

Heart rate (HR) and BP were measured weekly in conscious animals using a multi-channel noninvasive tail-cuff system as illustrated in the schematic in **Figure-1**. Briefly, each animal was placed in a restrainer attached to temperature-regulated BP-2000 platform. Subsequently, pre-calibrated manometer was attached to the tail of each animal to record the BP and HR. Average values of 10 consecutive recordings per animal were taken at each time point for comparison.

### Measurement of Pulmonary Arterial Pressure

Pulmonary arterial (PA) pressure was measured using an invasive technique by passing a catheter through the jugular vein into the PA. In brief, the animals were sedated by isoflurane inhalation, ventilated using the TOPO volume/pressure small animal ventilator (Kent Scientific Corp; Torrington, CT) and the jugular vein was exposed using surgical scalpel in order to insert a guiding catheter into the vein and then through the right atrium and ventricle into the PA for pressure measurements. The values were recorded in replicates and the average values were analyzed.

### Glucose Challenge Test

At 5-weeks following the initiation of LS/HS feeding, the animals were fasted overnight and baseline blood samples were collected from tail vein prior to challenging them with glucose solution (at 2 mg/g body weight) intraperitoneally. Blood samples were collected using heparinized catheters every 30 minutes until 150 minutes post-challenge and blood glucose concentration was measured using glucometer. Plasma was separated by centrifuging the samples at 3,000 rpm for 15 minutes and was stored at −80°C for insulin measurements.

### Measurement of Insulin Concentration

Plasma insulin concentration was measured using an ELISA colorimetric assay as per the supplier's recommendations. Background absorbance (read at 630 nm) was subtracted from the respective absorbencies (measured at 450 nm) and the sample plasma insulin concentration was calculated from a standard curve.

### Assessment of Insulin Sensitivity

The IR index, an indicator of insulin sensitivity, was calculated as described before [35]. In brief, the glucose values during the glucose challenge test above were averaged and expressed in mM. Area of the glucose curve was then calculated as described (60 min = 1 cm). The corresponding plasma insulin concentration was also averaged and expressed in µU/mL to calculate the area of the insulin curve. The IR index was then calculated by multiplying the two areas [35].

### Assessment of Renal Function

Renal function was assessed by measuring the volume of urine output; urinary creatinine and albumin levels. Metabolic cages were used to separate the feces from the urine and 24 hours urine volume was measured in each animal. Urinary albumin concentration was quantified using the Nephrat competitive ELISA assay following the supplier's recommendations. The sample albumin concentration was estimated from a standard curve and was expressed in mg/day after correlating with the 24 hours urine output. In addition, the urinary creatinine was quantified using the Creatinine Companion ELISA assay as per the instruction in the kit. Sample creatinine concentration was obtained from a standard curve and was expressed in mg/day. The urinary albumin-to-creatinine ratio (UACR) was calculated from the above measurements. Furthermore, renal fibrosis was assessed by the standard Masson's Trichrome stain.

### Measurement of serum ADMA and NO

The ADMA concentration in serum samples at baseline and 6-weeks post LS/HS-diet was measured using an ELISA assay as described [36]. In parallel, the NO levels of the baseline and 6-weeks samples were measured by ELISA as described by the manufacturer. ADMA and total NO (NOx) levels in the samples were calculated from the respective standard curves.

### Measurement of Tissue DDAH Activity

DDAH activity in liver tissue homogenates was measured using a stable isotope-based dilution assay as described [37]. In brief, about 50 mg of the harvested liver tissues were homogenized in PBS. Equal amounts of lysate were incubated with deuterium-labeled substrate (10 µM of [^2^H_7_]-ADMA) in a 96-well microplate for 1 hour at 37°C. Next, the reaction was stopped for determination of [^2^H_7_]-ADMA by LC-tandem MS in the presence of a double-isotope labeled ([^13^C_5_-^2^H_6_]-ADMA) internal standard. DDAH activity was calculated by subtracting the remaining [^2^H_7_]-ADMA from the amount added to the reaction and was expressed in nmol/g protein/min as described [37].

### Immunoblot Assay

After sacrificing the animals, tissues were harvested, snap frozen and homogenized for the preparation of total lysate. Protein concentration was measured using the Coomassie Plus BioRad assay. Liver lysates from the different groups were SDS-PAGE resolved and immunoblotted with anti- DDAH1 (Abcam) and JNK (Sigma) antibodies. Protein content was normalized to β-actin (ACTB; Sigma). Band intensities were compared using NIH's Image J analysis software (http://rsb.info.nih.gov/ij/docs/).

### Histological Examination and Fibrosis

For the histology, paraformaldehyde-fixed kidney sections were paraffin embedded and stained with H&E to assess tissue morphology. In addition, the kidney sections were also stained for fibrosis using Masson's Trichrome stain. Multiple fields were scanned microscopically by an expert renal pathologist who was unaware of the treatment groups. The degree of fibrosis was recorded as an estimate of the % of the renal cortex that was affected by interstitial fibrosis.

### Statistics

Statistical analysis to calculate the number of animals per study group was performed using an advanced power and sample size calculation (PS v3.0.14; Vanderbilt University). The study was designed to detect a difference in means (δ) of 0.2 with an estimated standard deviation (σ) of 0.18 at a significance level (α) of 0.05 with 85% power (β). All other statistical tests, unless stated otherwise, described in the study were performed using GraphPad Prism (La Jolla, CA). Comparison between two samples was performed using an unpaired student's t-test and multiple samples were compared using one-way ANOVA followed by Bonferroni posttest correction. Data was considered statistically significant at p value<0.05.

## Results

### The effect of INT-747 on high-salt diet induced hypertension

**Figure 2:**
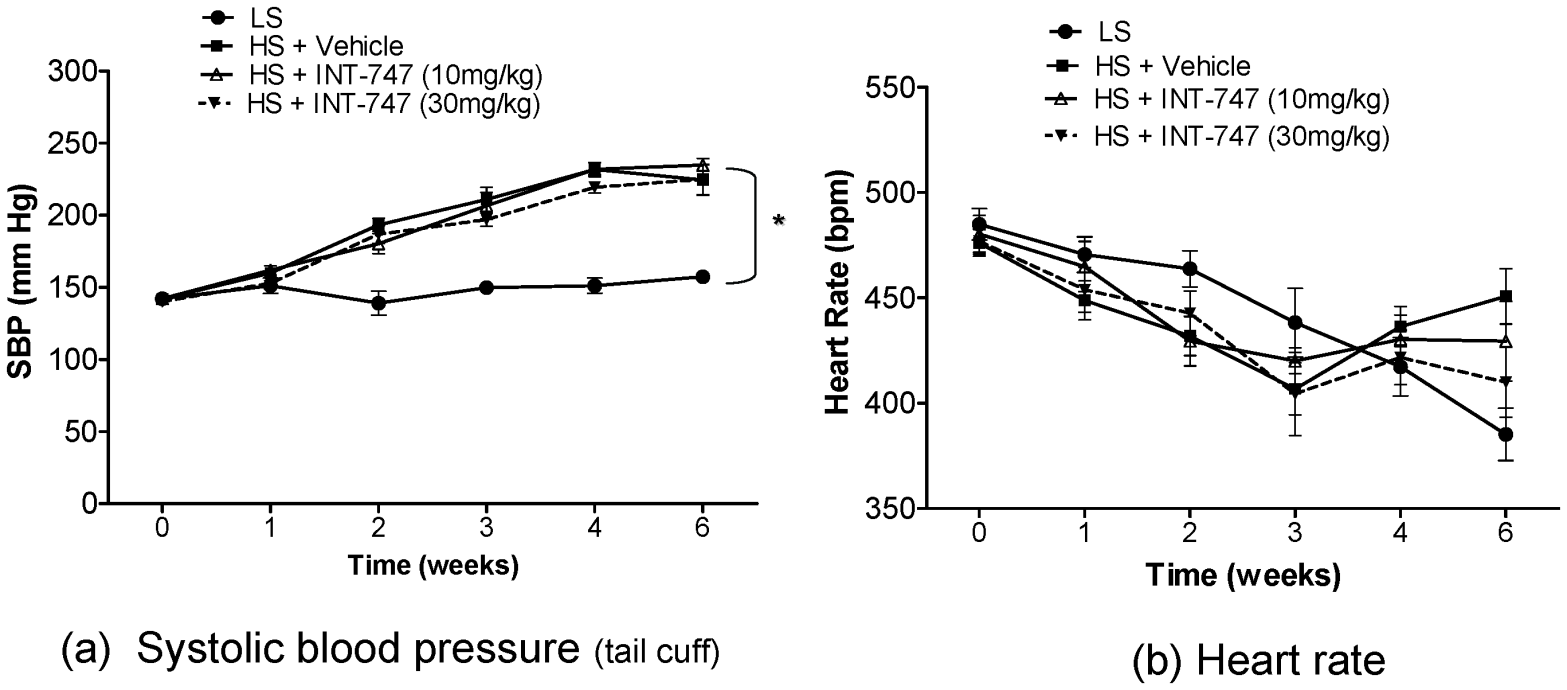
High-salt diet progressively increases systemic blood pressure. Weekly measurement of: a) systolic blood pressure (SBP) and b) heart rate (HR) by tail-cuff in Dahl salt-sensitive rats fed diet containing low salt (n = 9); high-salt and treated with vehicle (n = 15); high-salt and treated with 10 mg/kg/day INT-747 (n = 15) or high-salt and treated with high dose of INT-747 at 30 mg/kg/day (n = 15). Data is expressed as Mean±SEM. (*p<0.05 versus high-salt diet data. ANOVA followed by Bonferroni post-test).

A high-salt diet is known to increase mean arterial pressure (MAP) in salt-sensitive animals [38] and humans [22], [23]. Similarly, we observed that a HS-diet elevated systolic blood pressure (SBP) (**Figure-2a**). The SBP progressively increased over time reaching over 200 mm Hg after 4-weeks of HS-diet; significantly higher than that of LS animals (p<0.05). The treatment with INT-747 did not improve the BP. The HR was not statistically different between the groups (**Figure-2b**).

### The effect of high-salt diet on organ weight

The Dahl-SS animals are known to develop cardiac hypertrophy in response to HS-diet (http://www.harlan.com/). In this study, high-salt diet induced cardiac and renal hypertrophy, an effect that was not attenuated by INT-747 treatment (**Figure-S1**). In addition, pulmonary congestion was increased in the animals receiving HS and the lower dose of INT-747 (10 mg/kg) by comparison to the LS group (**Figure-S1**). By contrast, at the higher dose of INT-747 this effect was not observed, and there was also a trend towards lower pulmonary arterial (PA) pressure in this group (**Figure-S2**).

### The effect of INT-747 on renal function

**Figure 3:**
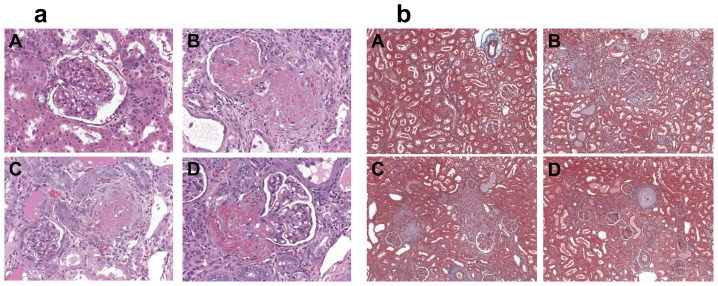
Assessing renal morphology and fibrosis. **a:** H&E staining to assess the renal morphology of Dahl rats following: LS-diet with nearly normal morphology (A) or HS-diet and administration of vehicle, showing fibrinoid arteriolar necrosis with extravasation of erythrocytes and thrombosis of glomerular capillaries (B); INT-747 at 10 mg/kg/day shows similar findings (C) as does INT-747 at 30 mg/kg/day (D) for 6 weeks. Representative images are shown. (400X mag). **b:** Masson's Trichrome staining to evaluate renal fibrosis (seen as blue-colored expansion of the interstitium between the tubules) in Dahl rat kidneys following: LS-diet (A) or HS-diet and administration of vehicle (B); INT-747 at 10 mg/kg/day (C) or INT-747 at 30 mg/kg/day (D) for 6 weeks. Representative images are shown. (100X mag).

In the salt-sensitive Dahl rat, a high-salt diet induces nephropathy as manifested by albuminuria. In our study, assessment of renal function showed that HS-diet profoundly impaired renal function as shown by significant increases in urinary albumin and creatinine values. In addition, the UACR analysis confirmed renal impairment in all the HS groups (**Figure-S3**). In addition, HS-diet increased kidney weight, an effect that was not regulated by INT-747 (**Figure-S1**). Furthermore, INT-747 did not improve renal pathology in the HS-fed animals. In all HS animals, severe thrombotic microangiopathy (TMA) was observed as well as fibrinoid necrosis of afferent arterioles with an onion-skin pattern of periarterial fibrosis and extravasation of erythrocytes (**Figure-3a**).

Evaluation of the corresponding Trichrome stained kidney sections showed tubular atrophy and interstitial fibrosis affecting 20–30% of the cortical area in the HS-diet fed animals compared to minimal (below 5%) tubular atrophy and fibrosis seen in the LS-diet group (**Figure-3b**).

### The effect of high-salt diet on DDAH protein expression and activity

**Figure 4:**
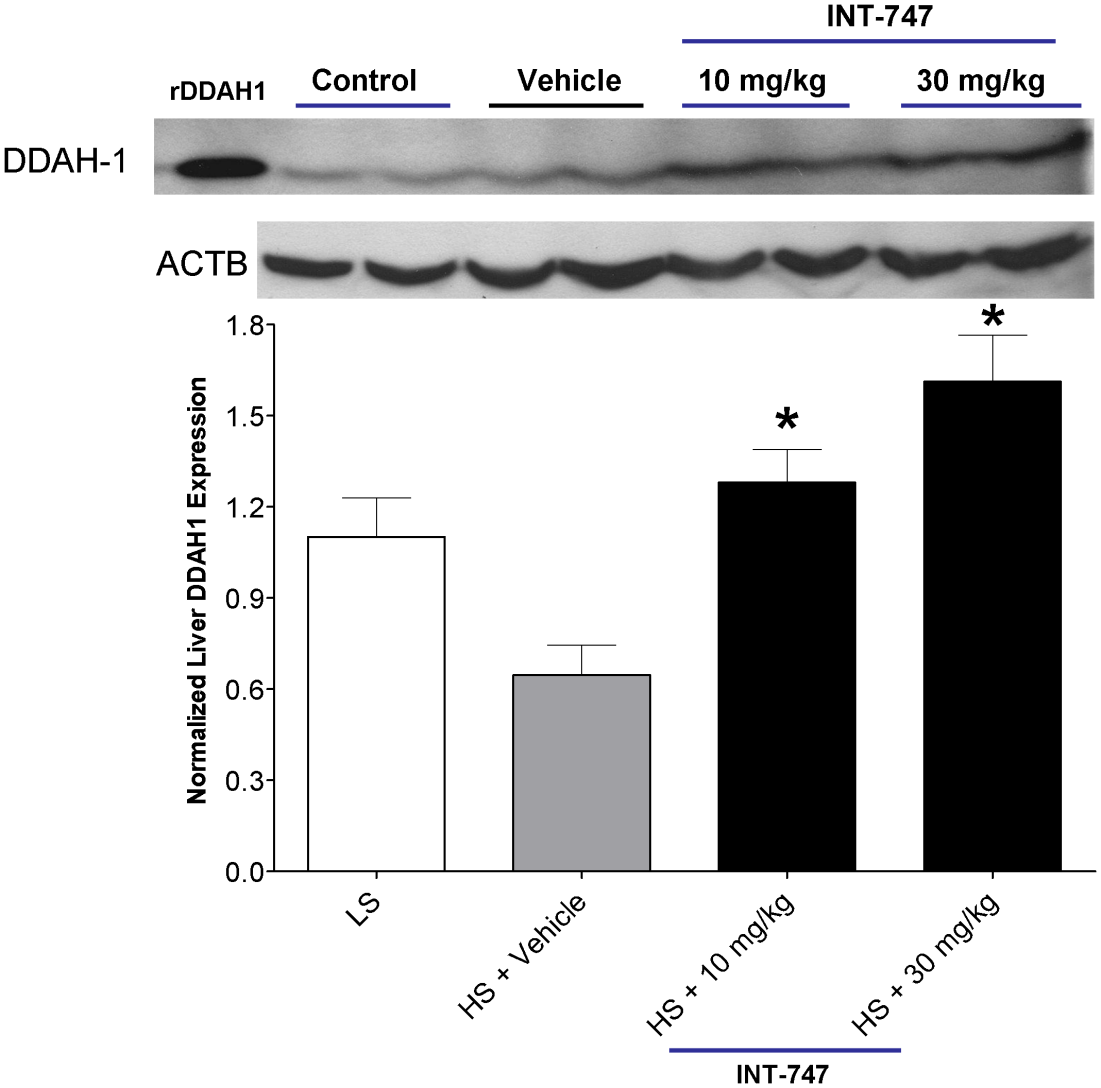
The effect of INT-747 treatment on DDAH1 expression in Liver. Animals were fed low (control)- or high-salt diet and treated with vehicle or INT-747 at 10 or 30 mg/kg/day for 6 weeks. Liver lysates were compared for DDAH1 expression by Western blot. rDDAH1: purified recombinant human DDAH1 described previously [53]. The DDAH1 expression was normalized to β-Actin (ACTB). DDAH = dimethylarginine dimethylaminohydrolase. Data is expressed as Mean±SEM. (*p<0.05 versus control value. ANOVA followed by Bonferroni post-test).

Our western blot analysis indicated that high-salt diet downregulated DDAH expression by almost 50% in the liver of HS-fed group receiving vehicle. By contrast, treatment with INT-747 reversed the salt-induced downregulation of DDAH expression (**Figure-4**). Despite the increase in liver DDAH protein expression following INT-747 treatment, liver DDAH activity of INT-747 group remained similar to the enzymatic activity in the tissues derived from the LS and vehicle groups (**Figure-S4**). It is possible that the oxidative stress that accompanies high-salt diet [39] could reduce DDAH expression and activity, as DDAH is highly sensitive to oxidative stress [27]. Such an effect on DDAH might offset the effect of INT-747 to increase DDAH expression.

In addition, INT-747 reduced expression of liver JNK-1 and JNK-2 (**Figure-S5**); proinflammatory proteins that may be upregulated by high-salt diet to interfere with normal insulin signaling [39].

**Figure 5:**
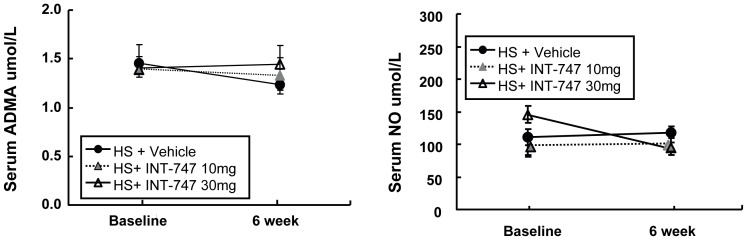
Measurements of serum ADMA and NO in Dahl rats at baseline and 6-weeks after feeding high-salt diet in the presence of vehicle or INT-747 treatment. NO and ADMA were measured as described in the text. Data is expressed as Mean±SEM. NO = nitric oxide; ADMA = asymmetric dimethylarginine.

### The effect of INT-747 on ADMA and NO concentration

Serum ADMA and NO concentration remained unchanged between the baseline and 6-weeks study period in the HS-fed animals despite the treatment group. Both ADMA and NO (NOx) levels were not favorably affected by treatment with INT-747 in this model (**Figure-5**).

### The effect of high-salt diet on insulin sensitivity

**Figure 6:**
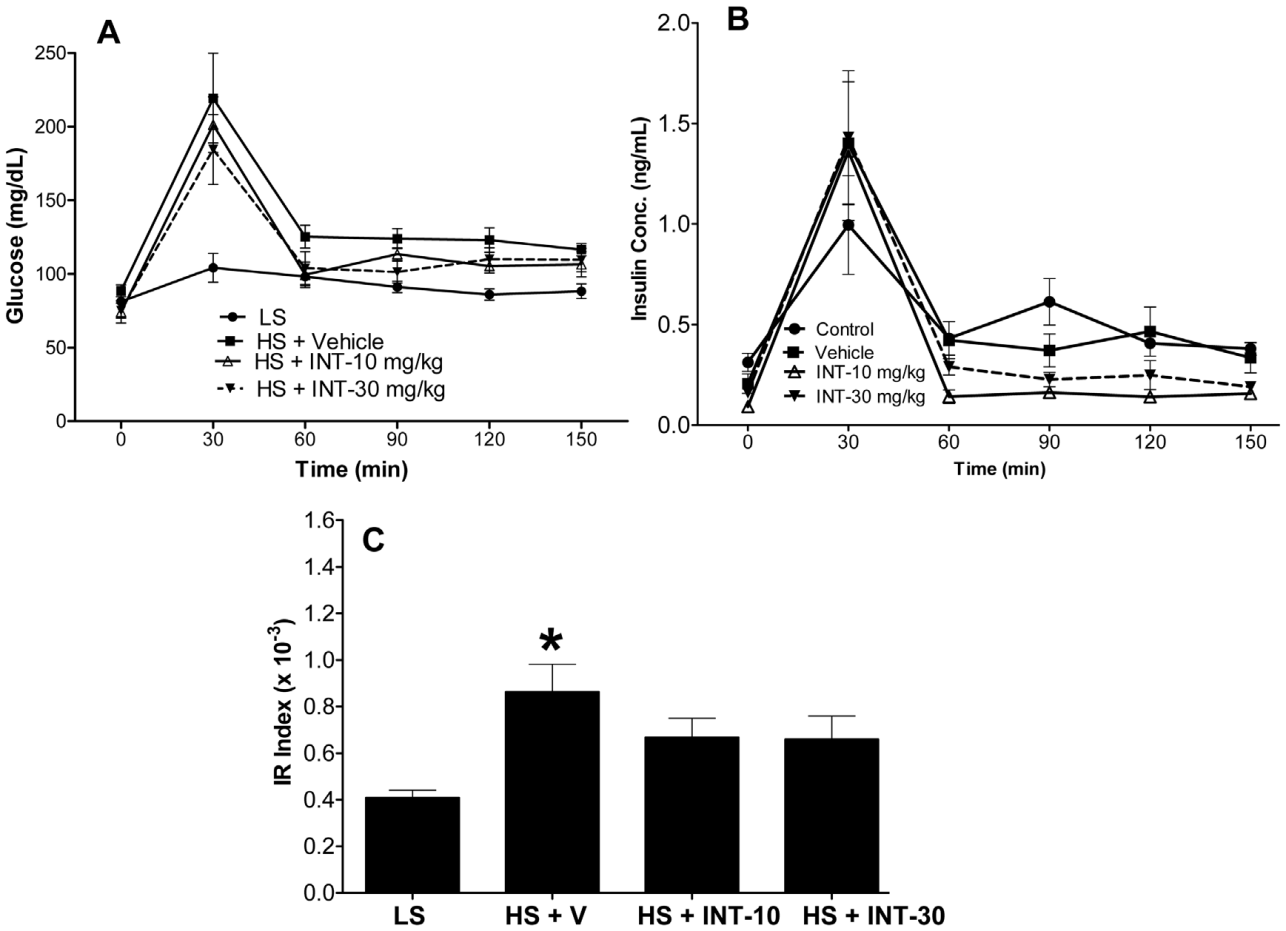
Blood glucose and insulin measurements to assess insulin sensitivity: Measurement of a) blood glucose \and b) plasma insulin concentration over time during GTT in Dahl rats fed with low or high-salt diet for 5-weeks prior to the glucose challenge test. Values are Mean ± SEM for: Control (n  = 6); Vehicle (n  = 7); INT-10 mg/kg/day (n  = 5) and INT-30 mg/kg/day (n = 9). *p<0.05 versus high-salt diet data. ANOVA followed by Bonferroni post-test. GTT = glucose tolerance test. In c), the effect of INT-747 treatment on insulin sensitivity is shown. Insulin Resistance (IR) index was calculated as described in the text. Data is expressed as Mean±SEM. (*p<0.05 versus low-salt diet data. ANOVA followed by Bonferroni post-test). LS = low salt; HS = high salt; V = vehicle; INT-10 = INT-747 at 10 mg/kg/day and INT-30 = INT-747 at 30 mg/kg/day.

In addition to being hypertensive, Dahl rats are known to be insulin resistant with an exacerbation in their IR after HS-feeding for about 4 weeks [39], [40]. In the current study, a glucose challenge increased blood glucose to a greater degree in HS animals, an effect which was blunted by INT-747 (**Figure-6a**). Similarly, after glucose challenge, there was a greater initial increase in insulin levels in the HS animals, which did not persist at later time points in the INT-747 group (**Figure-6b**).

The IR index was increased by HS-diet, (p<0.05), indicating deterioration in insulin sensitivity. This effect of HS-diet was reduced by INT-747; specifically the IR index was respectively reduced by 22.6% and 23.6% in the 10 mg/kg and 30 mg/kg INT-747 treated HS animals compared to the vehicle-treated HS animals, and was not statistically different compared to the LS-group (p>0.05). These results indicated that INT-747 improved insulin sensitivity (**Figure-6c**).

## Discussion

### Novelty and Significance:

The simultaneous effect of INT-747, a small molecule FXR agonist, on blood pressure and insulin sensitivity has not been studied before in Dahl Rats; an animal model that displays both systemic hypertension and IR. Our study addressed the scientific curiosity of whether it is possible to concurrently modulate both BP and insulin sensitivity using INT-747. We have also addressed the effect of high-salt diet on DDAH expression. Our key findings are: 1) INT-747 does not reduce systemic or pulmonary vascular pressure in high-salt fed Dahl rats. 2) INT-747 induces hepatic DDAH expression and enhances insulin sensitivity in this animal model as described below.

#### Role of DDAH/ADMA/NO in salt-sensitive hypertension and insulin resistance

There is mounting pharmacological and genetic evidence that implicate the NOS/ADMA/DDAH pathway in salt-sensitive HTN and IR. For example, studies have shown that high salt intake or increased salt retention impairs NO production [41], [42] and as a result endothelial function is blunted [43], causing an increase in mean arterial pressure (MAP) in pre-clinical models [38], [44] and in patients [45]. On the other hand, NO regulates salt-sensitivity [46] by blocking the entry of Na^+^/Cl^−^ into the thick ascending limb of the loop of Henle; a segment responsible for up to 30% of salt reabsorption [47], and by inhibiting the reabsorption of Na^+^ in cortical collecting ducts [48]. Furthermore, inhibition of NO using a NOS inhibitor reduces salt excretion, glomerular filtration rate (GFR) and diuresis [46], which is reversed by co-administration of the NO precursor L-arginine [49], [50], indicating the role of this pathway in vascular and renal pathophysiology. In addition, ADMA is elevated in patients with HTN and IR and is implicated in the progression of salt-sensitive HTN [21]–[23], [51]. In a preclinical study, overexpression of DDAH is associated with enhanced insulin sensitivity [28]. In addition, pharmacological manipulation of DDAH using FXR agonists is known to reduce ADMA [32], [34] and improve insulin sensitivity in humans (http://www.interceptpharma.com/) suggesting that DDAH may be an attractive target to improve insulin sensitivity [28], [52]. In fact, we have been carrying out efforts to discover and validate agents that enhance DDAH activity using the method we described [53].

#### INT-747 does not favorably influence tissue DDAH activity/circulating ADMA/NO levels, nor reverse salt-sensitive HTN

In the present study, we evaluated the pharmacological potential of INT-747 in regulating BP and insulin sensitivity in Dahl rats; a widely used pre-clinical model for HTN and IR [30], [31]. As an FXR agonist and an analogue of a primary human bile acid (chenodeoxycholic acid) and other cholic acid derivatives which are involved in modulating lipid, glucose and bile acid homeostasis, INT-747 has been clinically shown to improve liver function in patients with primary biliary cirrhosis (http://www.interceptpharma.com/) and is being evaluated for the treatment of nonalcoholic steatohepatitis (NASH) (http://www.interceptpharma.com/); a disease often associated with IR [54]. In our study, HS-diet significantly increased BP. Treatment with INT-747 did not significantly lower systemic or pulmonary arterial (PA) pressure. This finding may not be surprising in light of the lack of significant changes in tissue DDAH activity, circulating ADMA and NO levels following INT-747 treatment. In addition, we did not see significant INT-mediated improvement in the cardiac and renal hypertrophy that was caused by HS-diet feeding.

#### INT-747 effects on renal function

It is known that Dahl salt-sensitive animals develop nephropathy and albuminuria in response to HS- diet (http://www.harlan.com/). We found that a high-dose of INT-747 tended to improve renal function in HS animals as demonstrated by a reduction in albuminuria and urinary creatinine. Previous studies have demonstrated that INT-747 ameliorates nephropathy in animal models of diabetes (types I & II), associated with reductions in hyperlipoproteinemia, fibrosis, proteinuria, inflammation, and oxidative stress [55], [56]. Moreover, in 5/6 nephrectomized ApoE-deficient mice, INT-747 reduces chronic kidney disease (CKD)-induced vascular calcification independent of atherosclerosis progression [57]. However, our study of renal histology showed comparable TMA between the INT-747 and vehicle treated groups.

#### INT-747 favorably effects hepatic DDAH expression but not activity

More intriguingly, we demonstrate that HS-diet significantly downregulated hepatic DDAH1 expression (by about 50%). However, treatment with INT-747 protected the loss in hepatic DDAH1. Our observations are similar to those in an animal model of bile duct ligation (BDL) injury where treatment with INT-747 increases hepatic DDAH1 expression and improves portal pressure [34], [58]. Indeed, a small clinical study evaluating the therapeutic potential of INT-747 in regulating portal hypertension in patients with alcoholic cirrhosis has just completed (http://www.ukctg.nihr.ac.uk/trialdetails/ISRCTN22662520). Although INT-747 induced hepatic DDAH expression likely due to the presence of a putative FXR response element in the DDAH1 promoter [32], it did not favorably influence DDAH activity, nor did it favorably influence circulating ADMA and NO levels in this model.

#### INT-747 improves insulin sensitivity

Salt loading aggravates insulin resistance in Dahl rats; an animal model that inherently develops IR at weaning and prior to salt-loading [39], [40]. INT-747 enhanced insulin sensitivity in these animals as demonstrated by the reduction in IR index. It is possible that this effect is mediated in part by an increase in hepatic DDAH protein expression. However, the improvement in insulin sensitivity may be independent of any effect of INT-747 on DDAH. Indeed, it is likely that INT-747 enhances insulin sensitivity in part by regulating glucose homeostasis through other FXR-mediated effects [59]-[61]. Therefore, further mechanistic studies are justified to delineate the precise contribution of the DDAH pathway to bile acid-mediated glucose homeostasis system in this model. Meanwhile, data from a double blinded placebo controlled clinical study indicates that INT-747 improves insulin sensitivity (http://www.interceptpharma.com/) in nonalcoholic fatty liver disease (NAFLD) patients with type II diabetes.

#### Conclusion

The pharmacological potential of INT-747 was evaluated in an animal model of both dietary salt-induced hypertension and IR. This study reveals that it is unlikely for INT-747 to be a dual- or poly- pharmacologic agent for the treatment of systemic hypertension or pulmonary hypertension and IR. Our study provides a valuable insight in that INT-747 may not be simultaneously used as an insulin sensitizer and antihypertensive agent. However, it has a promising potential to enhance insulin sensitivity in hypertensive patients.

## Supporting Information

Figure S1 **Measurement of organ weights for: a) heart b) lungs c) liver, and d) kidneys.** Data is normalized organ weight to the respective body weight at time of sacrifice. Lungs and kidneys weight was combined total weight for the right and left tissues. Data is expressed as Mean±SEM. (*p<0.05 versus low-salt diet data. ANOVA followed by Bonferroni post-test).
(TIF)

Figure S2 **Assessing the effect of INT-747 on Pulmonary Arterial (PA) pressure.** Animals were intubated and catheter was inserted into the PA for pressure measurement in low salt, vehicle, INT-747 (10 mg/kg/day) and INT-747 (30 mg/kg/day) groups. Data is expressed as Mean±SEM.
(TIF)

Figure S3 **Assessment of renal function by measuring urinary creatinine and albumin.** The urinary creatinine and albumin values were normalized to 24 h urine output and the urinary-albumin-to-creatinine-ratio (UACR) was calculated as described in the text. Data is expressed as Mean±SEM. (*p<0.05 versus low-salt diet data. ANOVA followed by Bonferroni post-test).
(TIF)

Figure S4 **Assessment of tissue DDAH activity using a stable-isotope assay in liver lysates of Dahl rats following: LS-(n = 9) or HS- diet and administration of vehicle (n = 8) or INT-747 at 10 mg/kg/day (n = 8) or at 30 mg/kg/day (n = 9) for 6 weeks.** Data is from triplicate experiments and is expressed as Mean±SEM. (p>0.05 among all groups. ANOVA followed by Bonferroni post-test).
(TIF)

Figure S5 **The effect of INT-747 treatment on the expression of c-JNK isoforms 1 and 2 in the Liver.** Animals were fed low (control)- or high-salt diet and treated with vehicle or INT-747 at 10 or 30 mg/kg/day for 6 weeks. Liver lysates were compared for c-JNK1 and 2 expression by Western blot. The c-JNK expression was normalized to β-Actin (ACTB). C-JNK = c-Jun N-terminal Kinase. Data is expressed as Mean±SEM. (*p<0.05 versus the data of low-salt or high-salt diet and INT-747 treated with either dose. ANOVA followed by Bonferroni post-test).
(TIF)
