# Supplementary figures and images for: FXR Agonist INT-747 Upregulates DDAH Expression and Enhances Insulin Sensitivity in High-Salt Fed Dahl Rats
Source: PLoS One. 2013 Apr 4;8(4):e60653. doi: 10.1371/journal.pone.0060653 (PMC3617194; doi:10.1371/journal.pone.0060653)

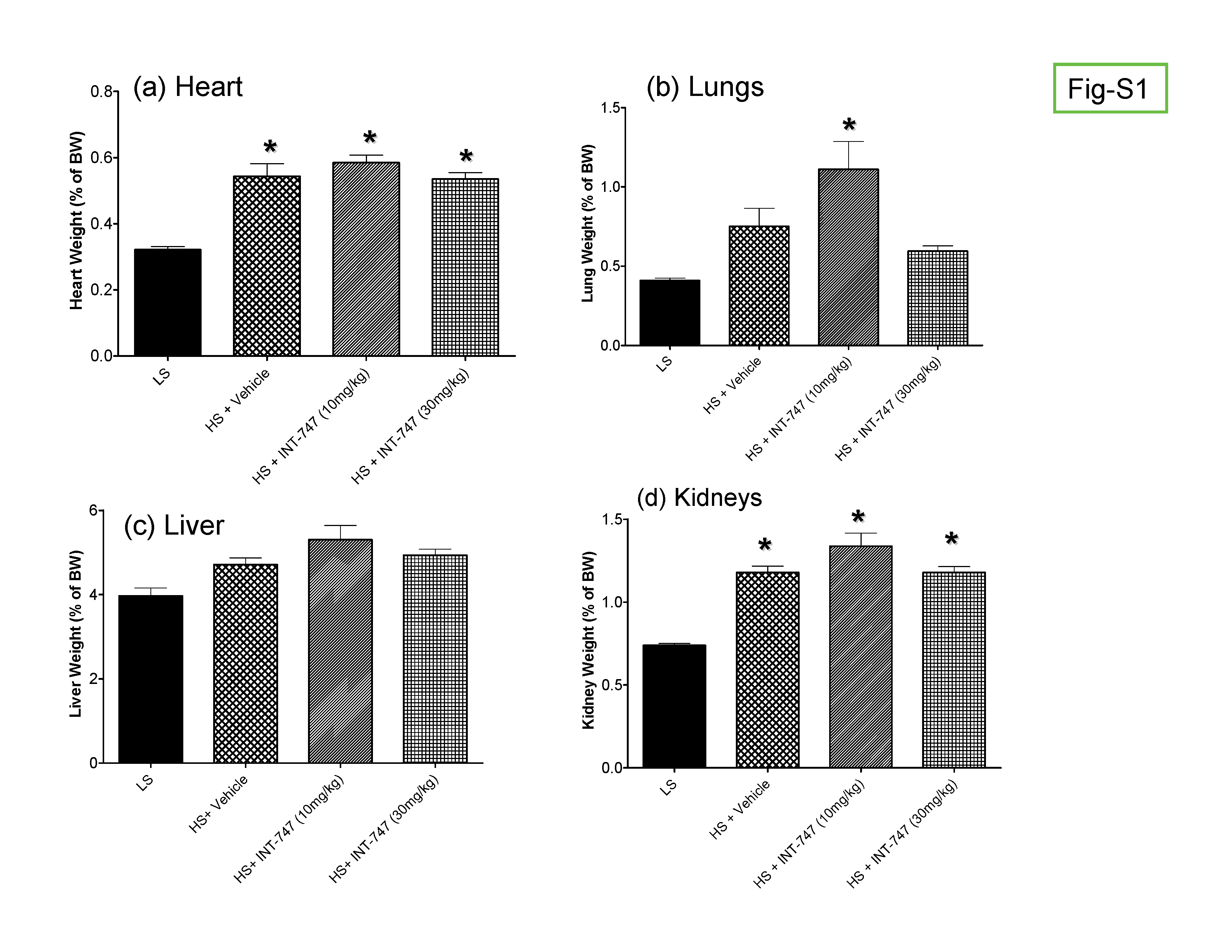

Supplement: Figure S1 — Measurement of organ weights for: a) heart b) lungs c) liver, and d) kidneys. Data is normalized organ weight to the respective body weight at time of sacrifice. Lungs and kidneys weight was combined total weight for the right and left tissues. Data is expressed as Mean±SEM. (*p<0.05 versus low-salt diet data. ANOVA followed by Bonferroni post-test). (TIF) [file pone.0060653.s001.tif]

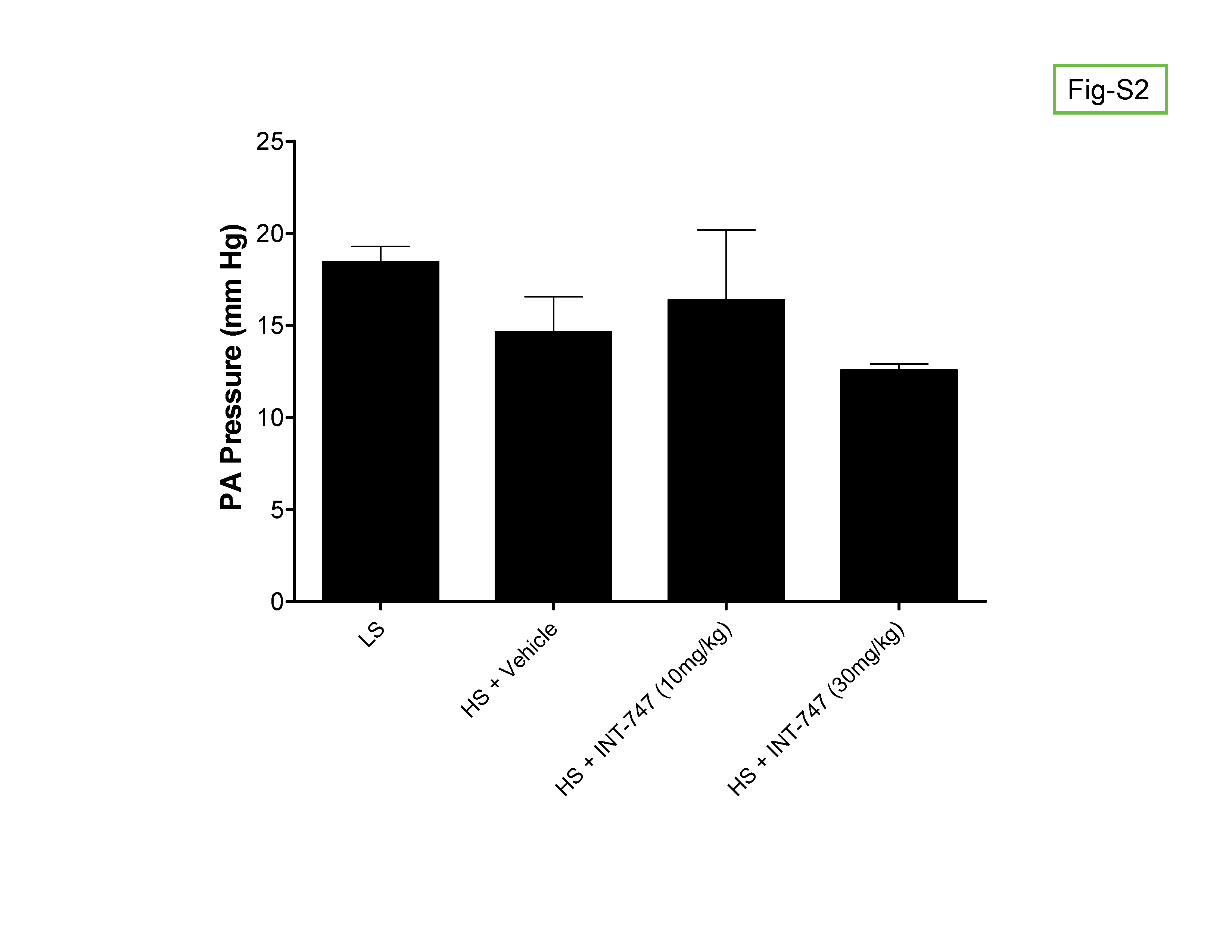

Supplement: Figure S2 — Assessing the effect of INT-747 on Pulmonary Arterial (PA) pressure. Animals were intubated and catheter was inserted into the PA for pressure measurement in low salt, vehicle, INT-747 (10 mg/kg/day) and INT-747 (30 mg/kg/day) groups. Data is expressed as Mean±SEM. (TIF) [file pone.0060653.s002.tif]

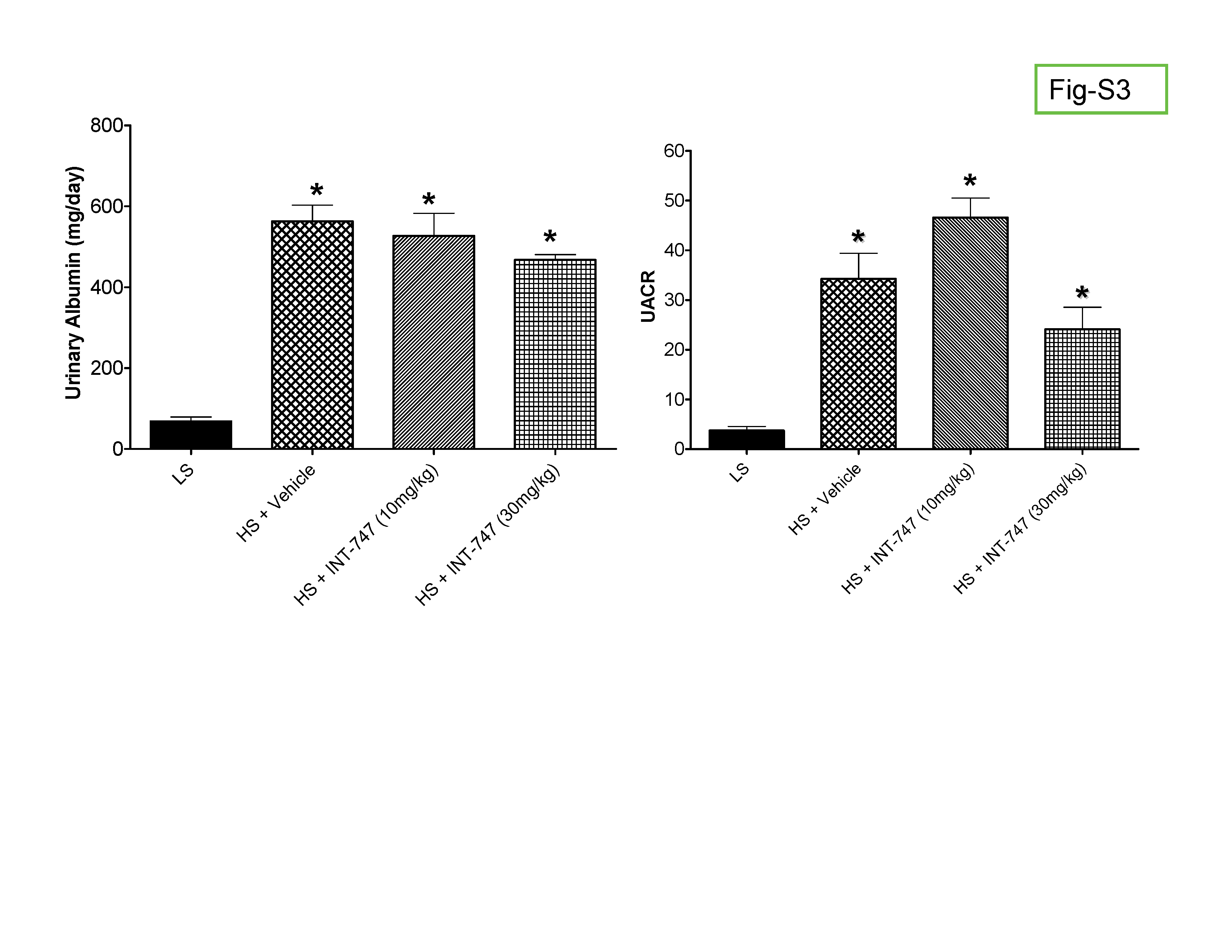

Supplement: Figure S3 — Assessment of renal function by measuring urinary creatinine and albumin. The urinary creatinine and albumin values were normalized to 24 h urine output and the urinary-albumin-to-creatinine-ratio (UACR) was calculated as described in the text. Data is expressed as Mean±SEM. (*p<0.05 versus low-salt diet data. ANOVA followed by Bonferroni post-test). (TIF) [file pone.0060653.s003.tif]

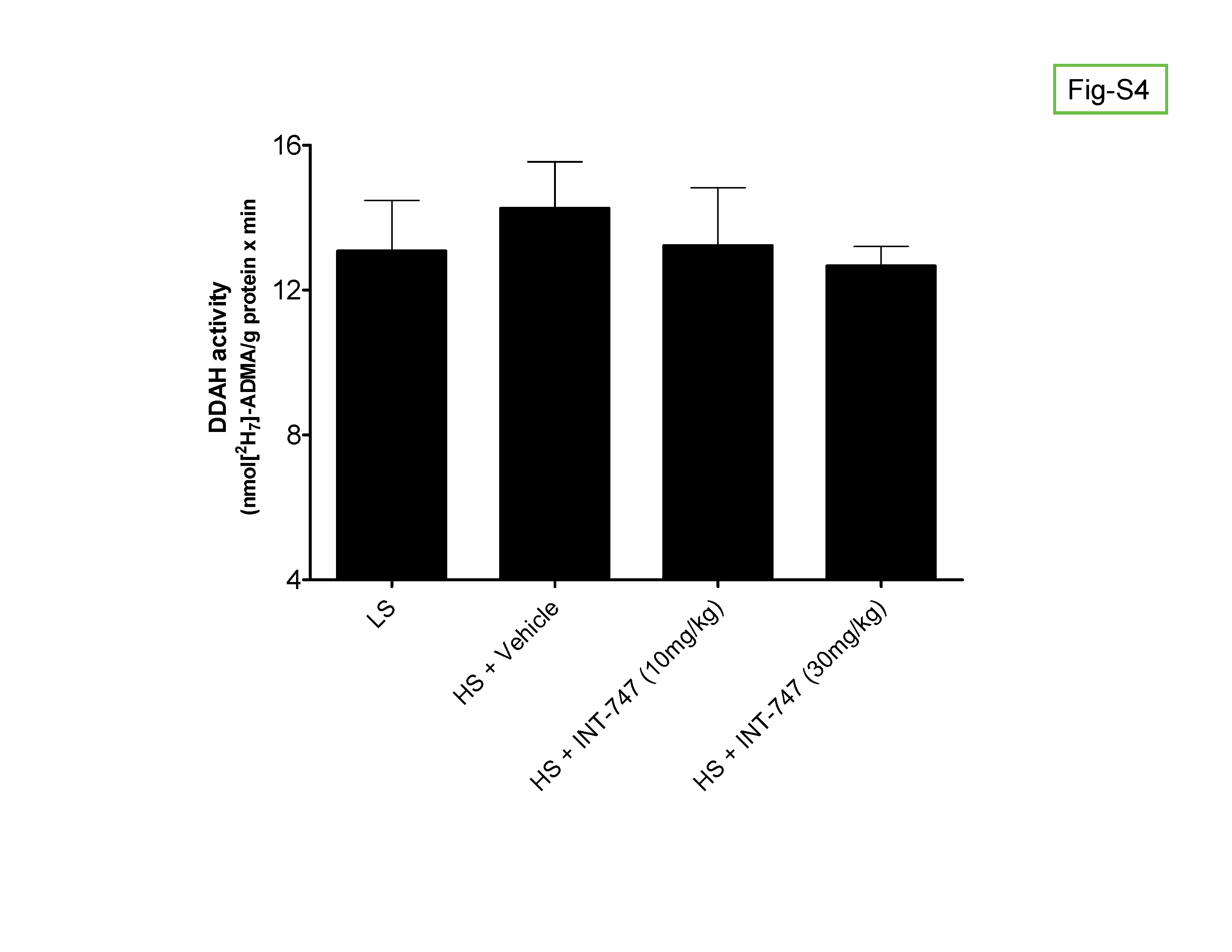

Supplement: Figure S4 — Assessment of tissue DDAH activity using a stable-isotope assay in liver lysates of Dahl rats following: LS-(n = 9) or HS- diet and administration of vehicle (n = 8) or INT-747 at 10 mg/kg/day (n = 8) or at 30 mg/kg/day (n = 9) for 6 weeks. Data is from triplicate experiments and is expressed as Mean±SEM. (p>0.05 among all groups. ANOVA followed by Bonferroni post-test). (TIF) [file pone.0060653.s004.tif]

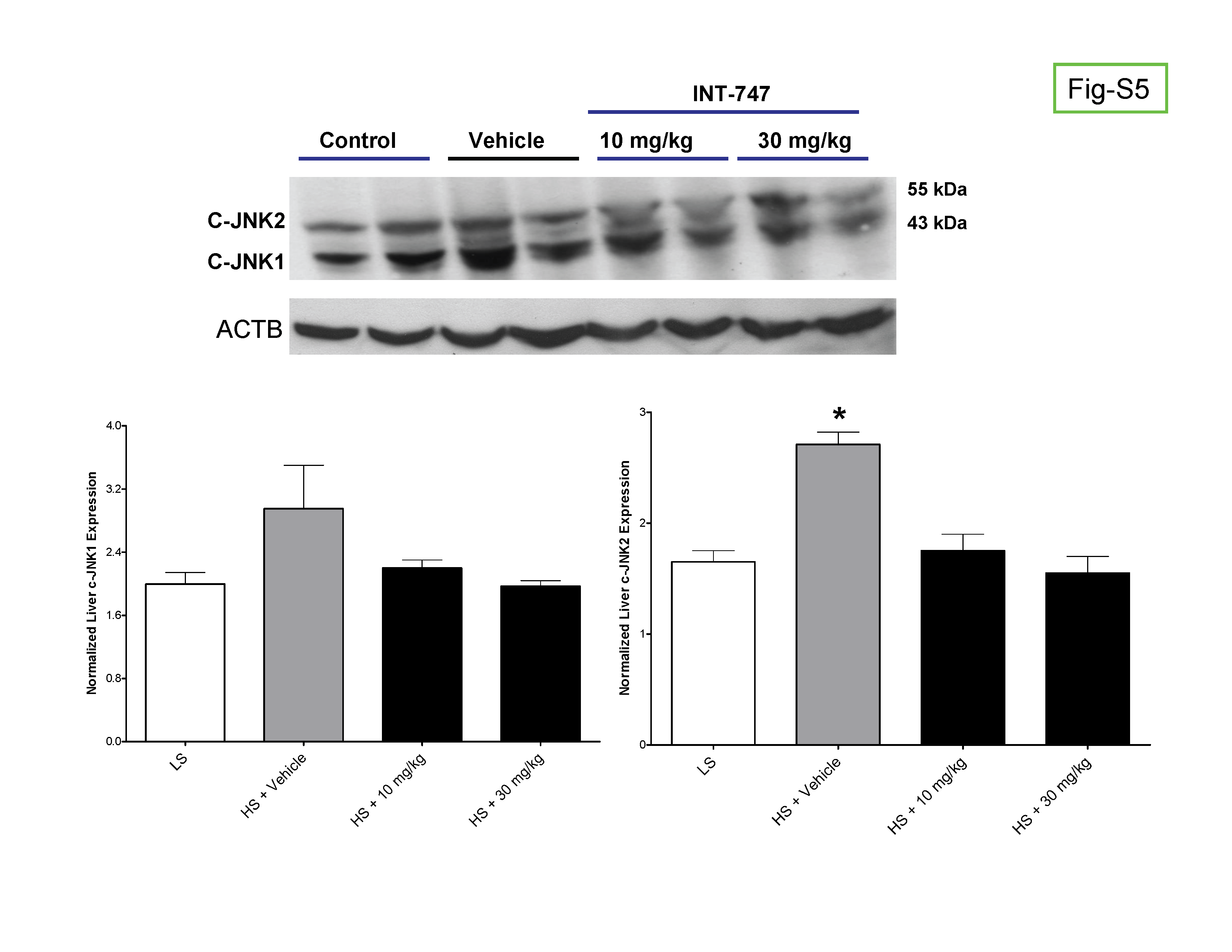

Supplement: Figure S5 — The effect of INT-747 treatment on the expression of c-JNK isoforms 1 and 2 in the Liver. Animals were fed low (control)- or high-salt diet and treated with vehicle or INT-747 at 10 or 30 mg/kg/day for 6 weeks. Liver lysates were compared for c-JNK1 and 2 expression by Western blot. The c-JNK expression was normalized to β-Actin (ACTB). C-JNK = c-Jun N-terminal Kinase. Data is expressed as Mean±SEM. (*p<0.05 versus the data of low-salt or high-salt diet and INT-747 treated with either dose. ANOVA followed by Bonferroni post-test). (TIF) [file pone.0060653.s005.tif]
